# Supplementary material for: Circular and Fusion RNAs in Medulloblastoma Development
Source: Cancers (Basel). 2022 Jun 26;14(13):3134. doi: 10.3390/cancers14133134 (PMC9264760; doi:10.3390/cancers14133134)

Supplementary File S8

A. Genomic region encompassing TRAM1 and NCOA2 according to NCBI. Positions of fusion junction are in red and blue.

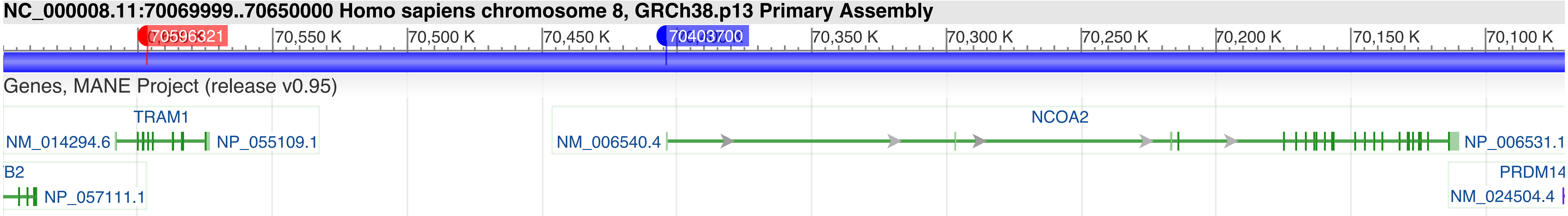

B. Genomic region encompassing ADAMTSL3 and SH3GL3 according to NCBI. Positions of fusion junction are in red and blue.

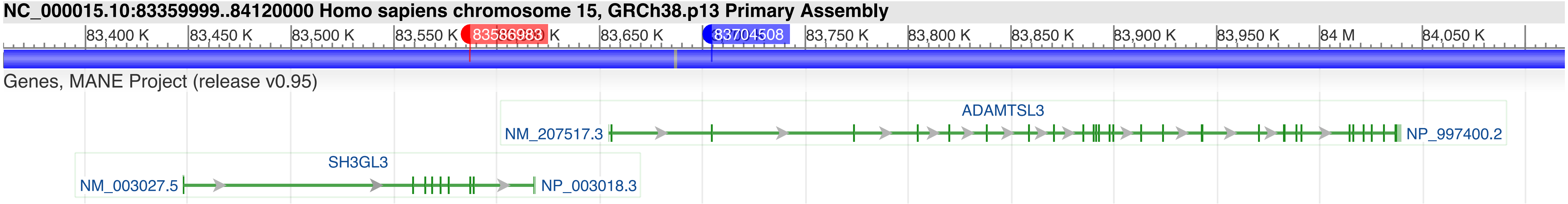

C. Genomic region encompassing ARL17A, ARL17B and KANSL1 according to NCBI. Positions of fusion junctions are in red, green and blue.

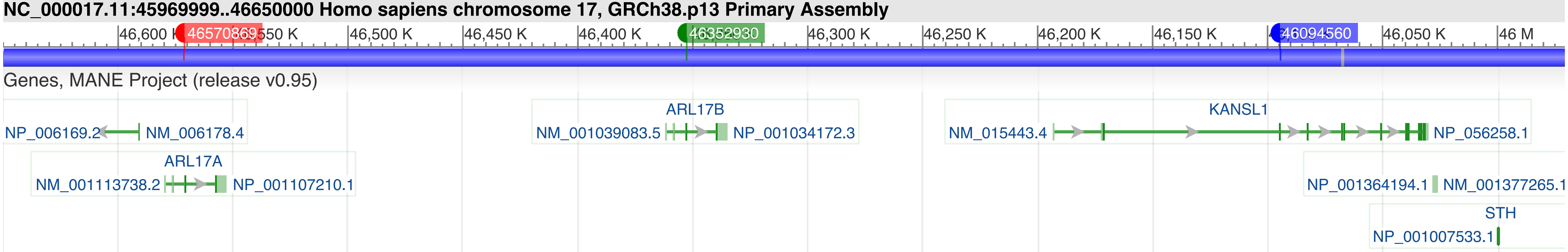

D. Genomic region encompassing ADGRG7 and TFG according to NCBI. Positions of fusion junction are in red and blue.

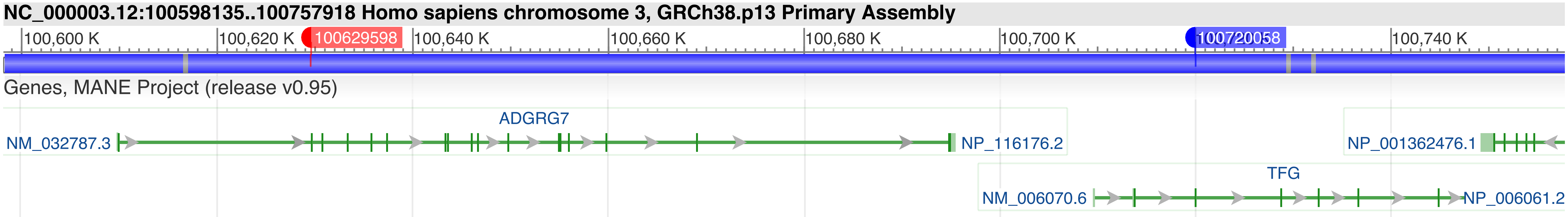

E. Genomic region encompassing CASC8 and PVT1 according to NCBI. Positions of fusion junction are in red and blue.

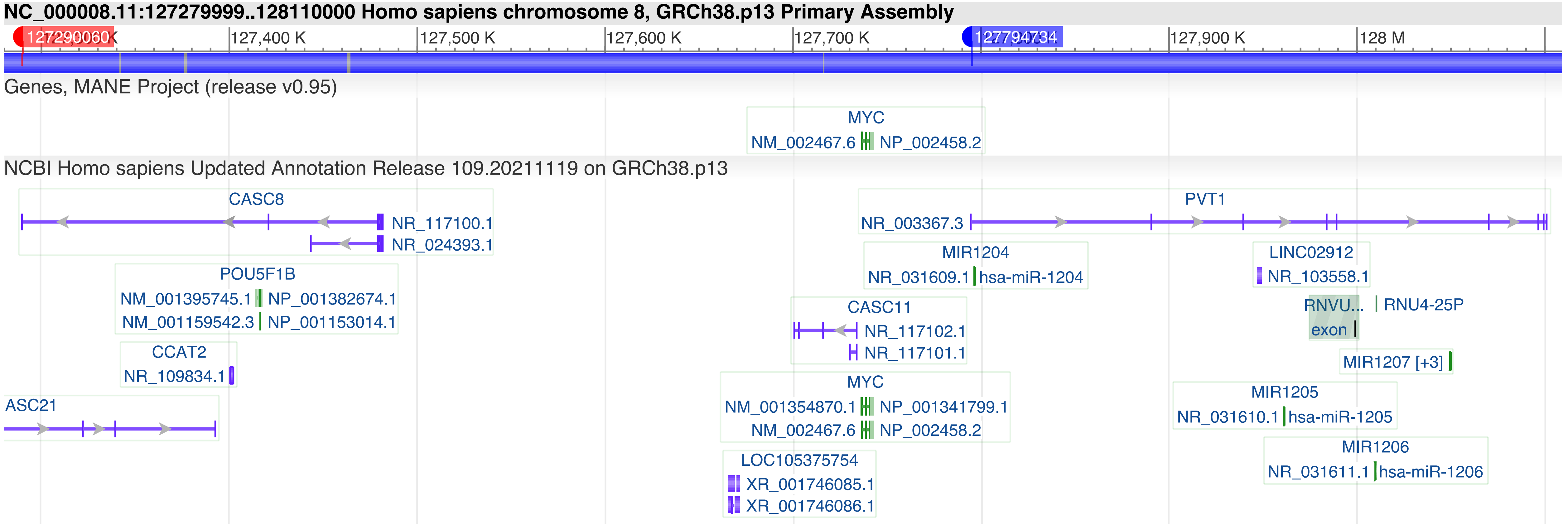

Supplement: Supplementary file 1 [file cancers-14-03134-s001.zip › File S8_Genomic organization of constituent genes for selected fusion RNAs.pdf]
