# Supplementary material for: Circular and Fusion RNAs in Medulloblastoma Development
Source: Cancers (Basel). 2022 Jun 26;14(13):3134. doi: 10.3390/cancers14133134 (PMC9264760; doi:10.3390/cancers14133134)
Supplement: Supplementary file 1 [file cancers-14-03134-s001.zip › Supplementary figure legends.pdf]

Supplementary figure legends

Figure S1. Differentially expressed mRNAs in human medulloblastoma and normal cerebellum. Principal component analysis (PCA) of the variance stabilized DESeq2 data from the (**A**) mRNA and (**B**) circular RNA preparations. (**C**) Volcano plots of the RNA-seq data from the SHH, WNT, Group 3 and Group 4 medulloblastoma tumor and normal cerebellum samples. Cutoff thresholds and annotated selected mRNAs are defined as in Figure 1A.

Figure S2. Expression of the selected circular RNAs from SHH, WNT, Group 3 and Group 4 medulloblastoma. Violin plots of the expression of the selected circular RNAs (circRNA) (Figure 1A) in SHH (**A**)**,** WNT (**B**), Group 3 (**C**) and Group 4 (**D**) medulloblastoma (upper panels). The expression of the respective linear mRNAs is also shown in the lower panels, respectively. The plots are sorted by the abundance of the circular RNA counts.

Figure S3. Differentially expressed circular RNAs in each medulloblastoma subtype compared to the remaining subtypes and cerebellum. Volcano plots of the RNA-seq data of each of the SHH, WNT, Group 3 and Group 4 medulloblastoma subtypes in comparison to the other subtypes and cerebellum (Rest). Cutoff thresholds are assessed with the DESeq2 method, where the Wald significance test is applied as $|\log2 \text{ fold change}| > 1$, $padj < 0.05$. Annotated are selected circular RNAs defined as the top 5 with highest normalized mean count across samples, the top 5 with lowest padj value, the top 5 with highest log2 fold change and the top 5 with lowest log2 fold change.

Figure S4. Differentially expressed mRNA and circular RNAs in progressive relative to initial SHH tumors. Principal component analysis (PCA) of the variance stabilized DESeq2 data from the (**A**) mRNA and (**C**) circular RNA preparations. (**B, D**) Volcano plots of the RNA-seq data from the progressive and initial SHH medulloblastoma samples for mRNAs and circular RNAs, respectively. Cutoff thresholds and annotated selected mRNAs are defined as in Figure 1A, except in the case of circular RNAs where the threshold is relaxed to $p < 0.05$ and $|\log2FoldChange| > 1$.

Figure S5. Top 20 fusion transcripts in linear and circular RNAs from cerebellum and medulloblastoma. Box plots of the top 20 highest expressed linear and circular fusion transcripts in cerebellum (mdata CB and cdata CB) and medulloblastoma (mdata MB and cdata MB). For each fusion, the fraction of counts of that transcript within a sample is presented as % of total counts. Outliers that differ significantly from the rest of the dataset are plotted as individual points beyond the whiskers on the box plot.

Figure S6. Original gel electrophoresis for Figure 3. The original gel run for the electrophoresis experiment of Figure 3 is shown.
