# Supplementary material for: Circular and Fusion RNAs in Medulloblastoma Development
Source: Cancers (Basel). 2022 Jun 26;14(13):3134. doi: 10.3390/cancers14133134 (PMC9264760; doi:10.3390/cancers14133134)

Figure S1

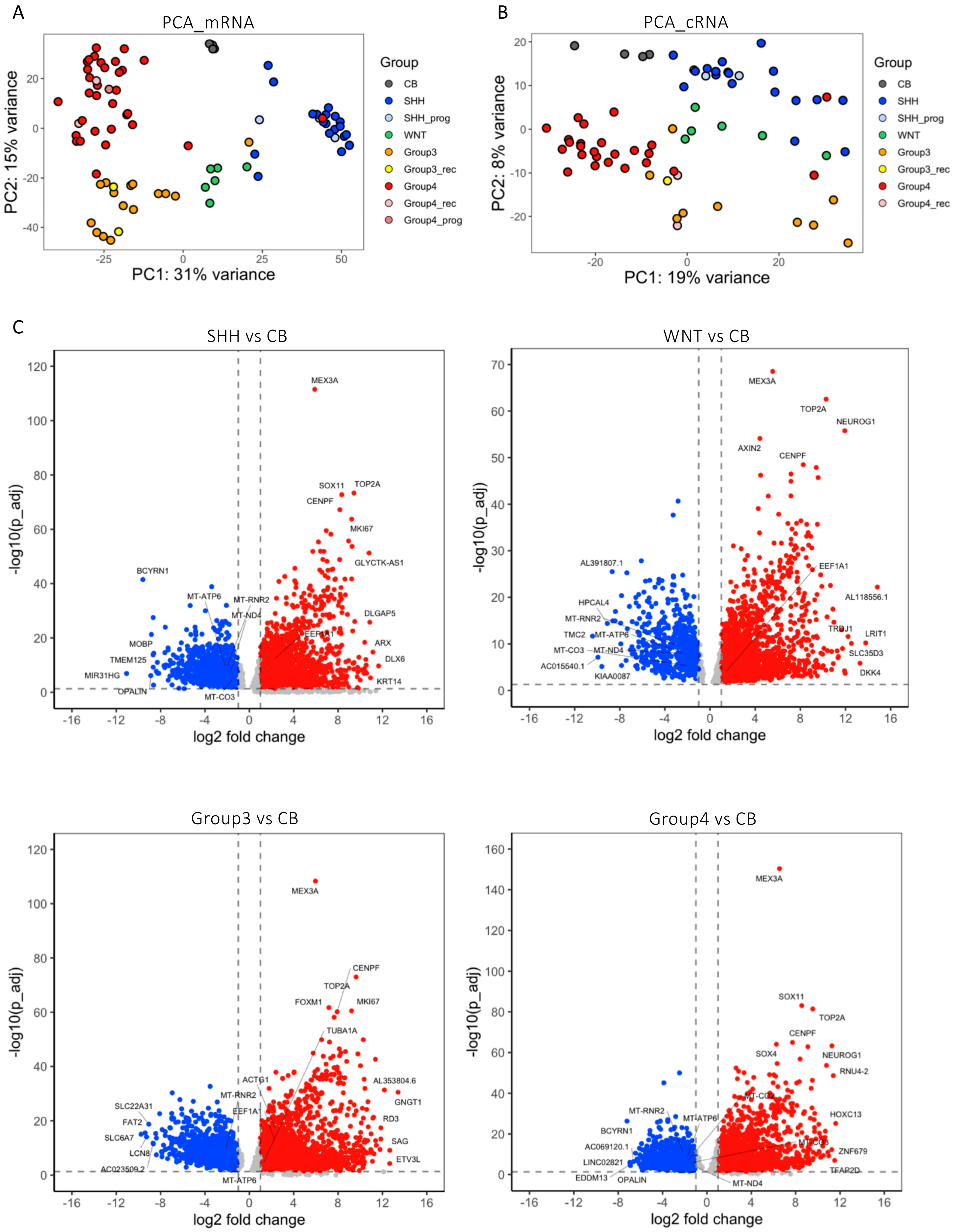
A
PCA_mRNA
PC1: 31% variance
PC2: 15% variance
Group
CB
SHH
SHH_prog
WNT
Group3
Group3_rec
Group4
Group4_rec
Group4_prog
B
PCA_cRNA
PC1: 19% variance
PC2: 8% variance
C
SHH vs CB
WNT vs CB
Group3 vs CB
Group4 vs CB
log2 fold change
-log10(p_adj)
MEX3A
TOP2A
SOX11
CENPF
MKI67
NEUROG1

Figure S2A

SHH_circRNA

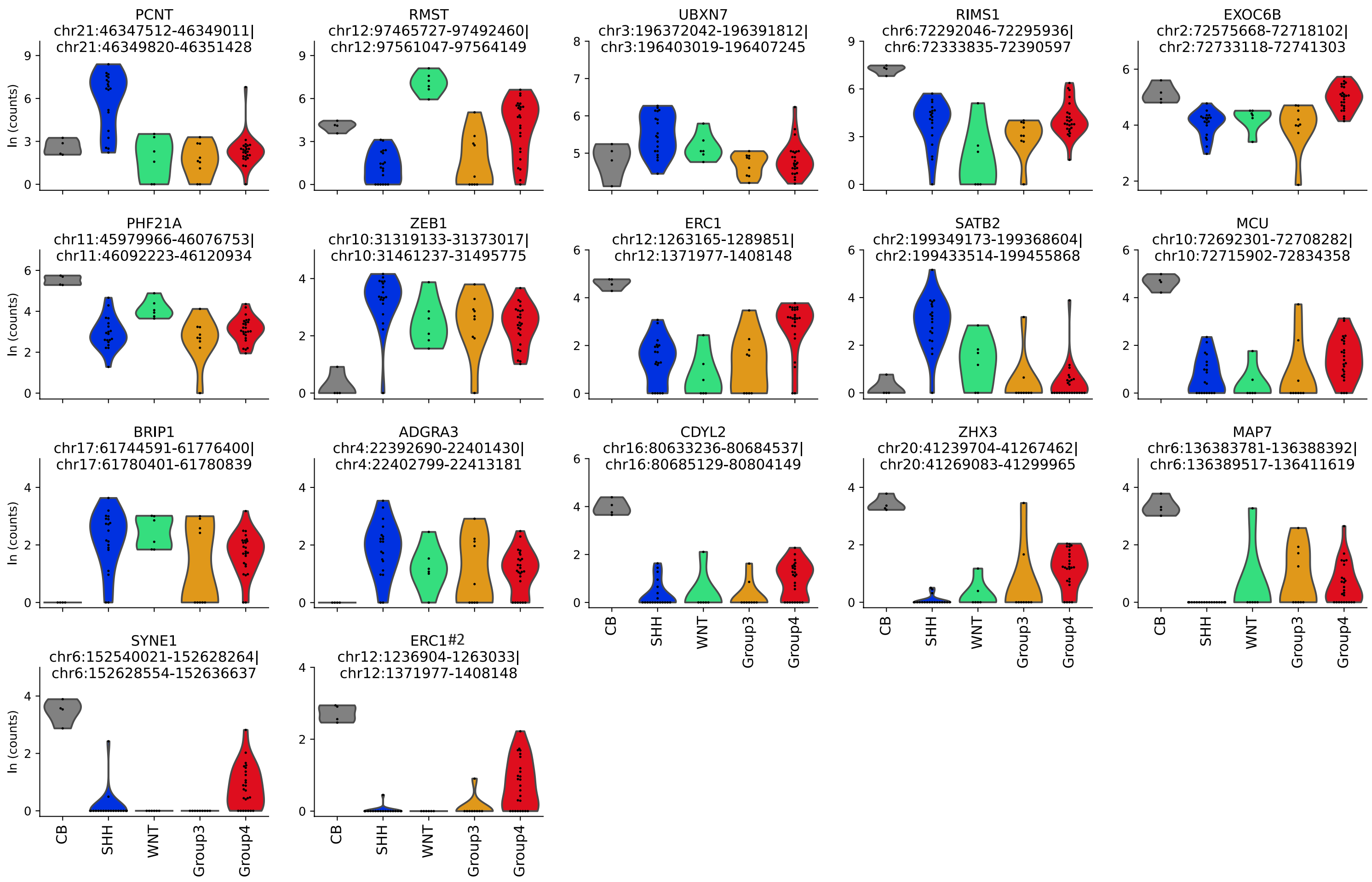

SHH_mRNA

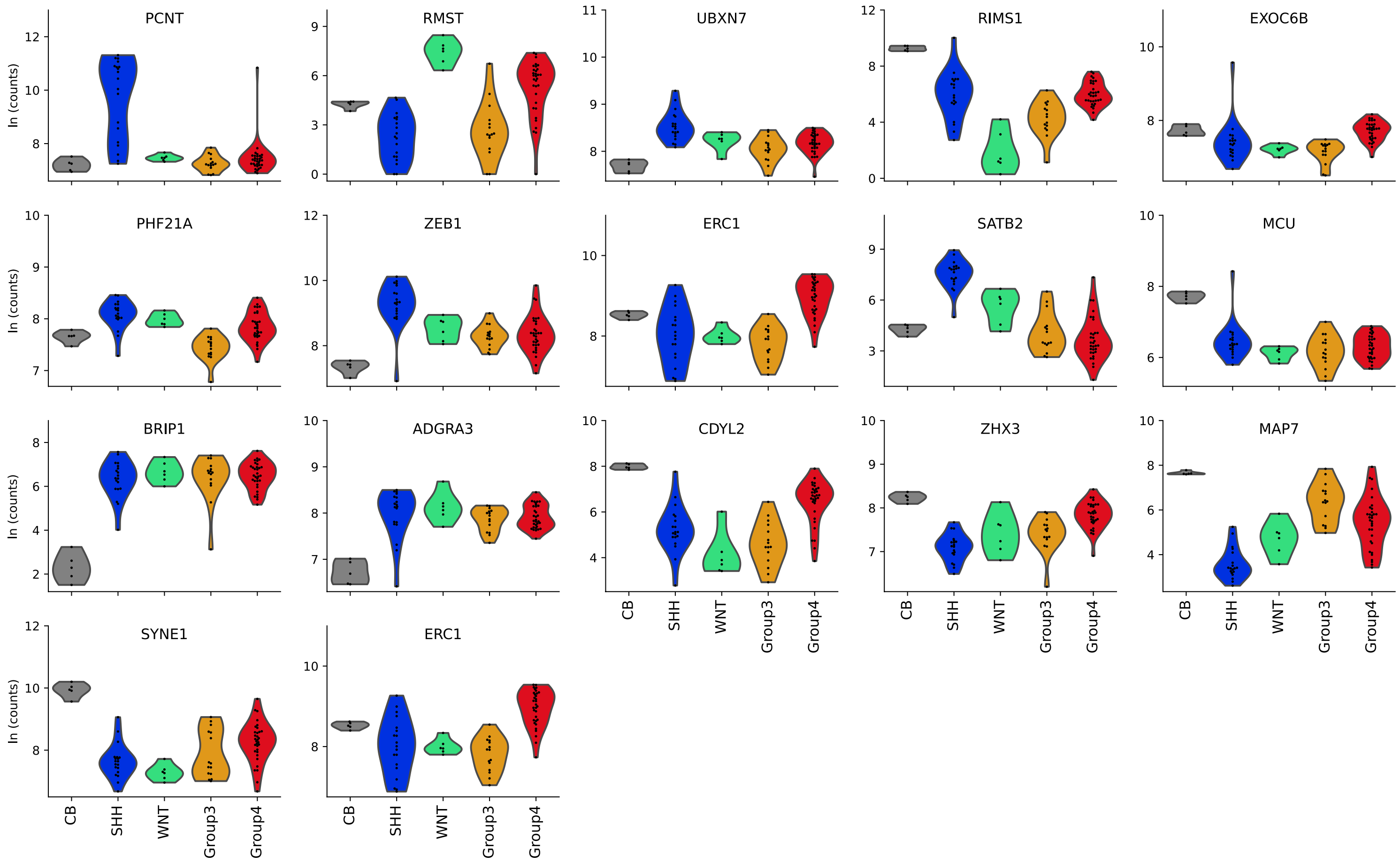

Figure S2B

WNT_circRNA

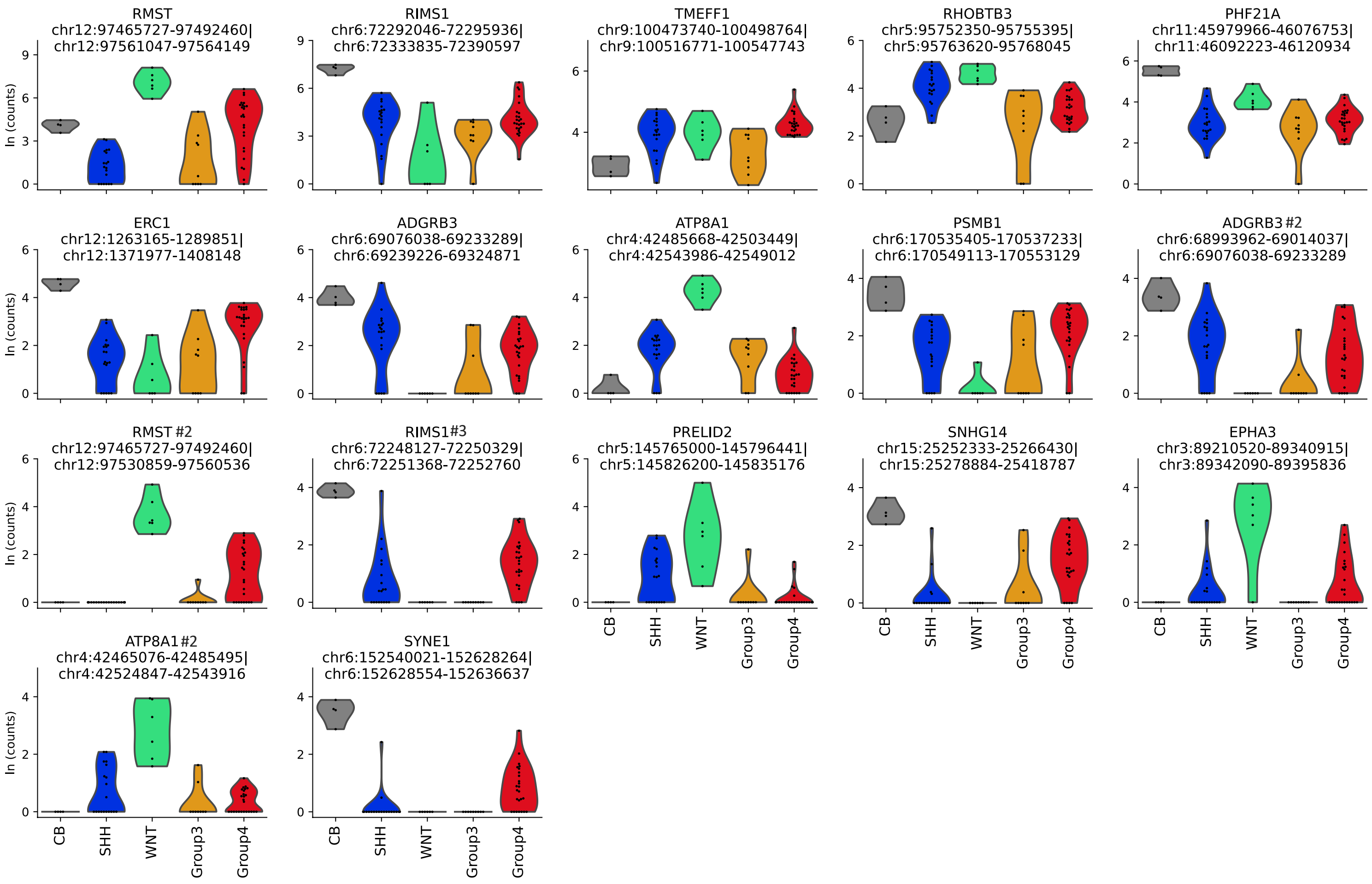

WNT_mRNA

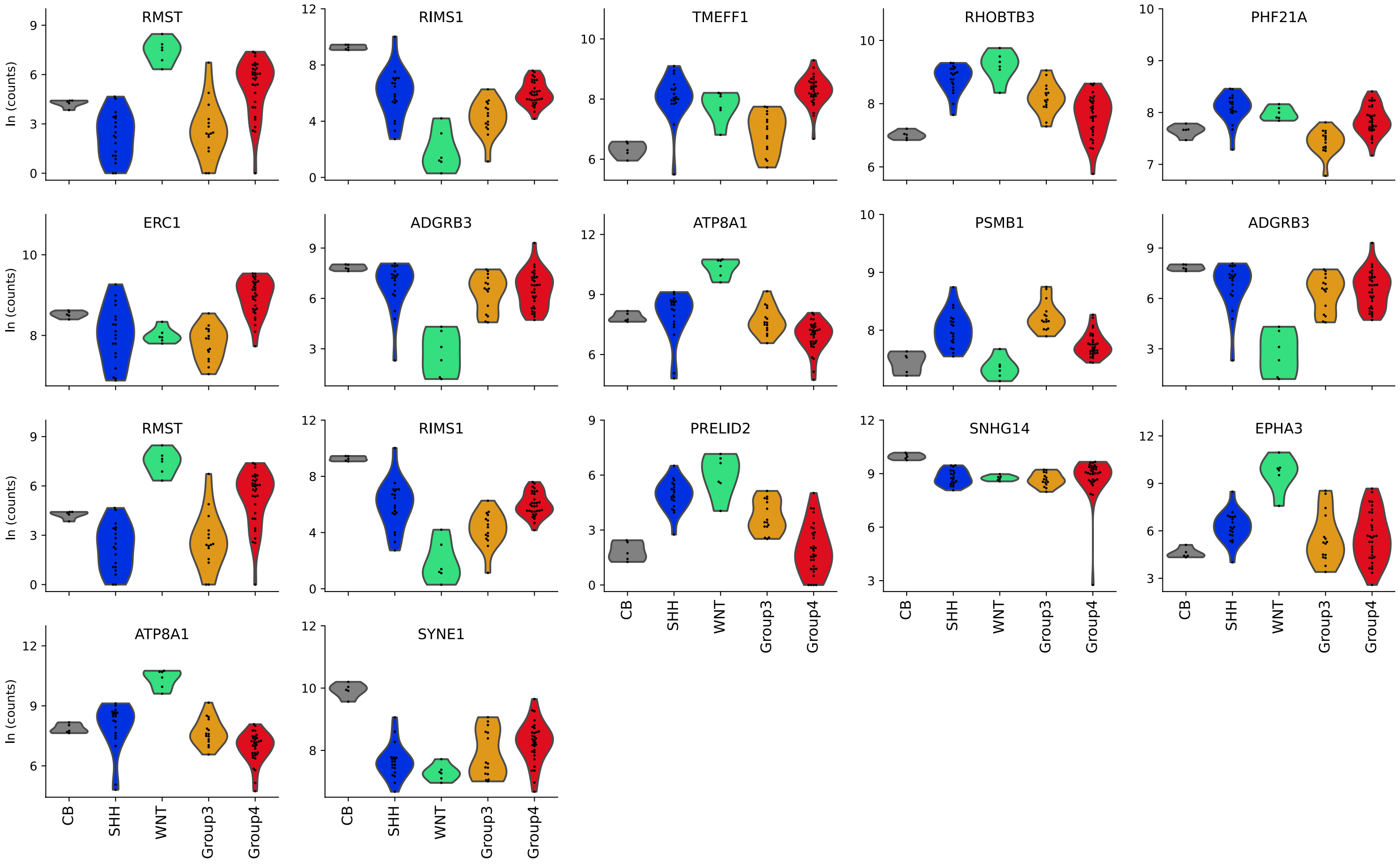

Figure S2C

Group3_circRNA

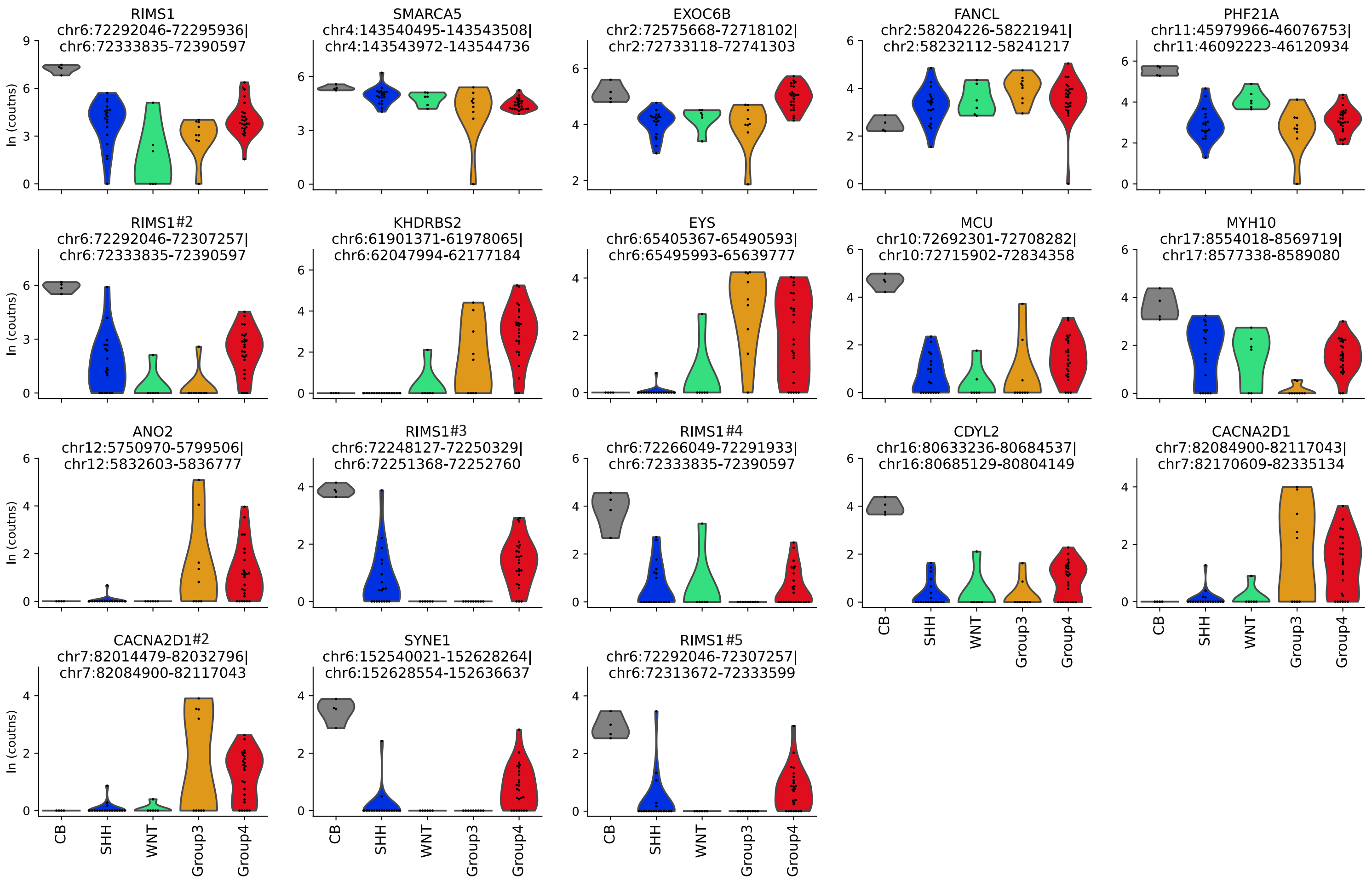

Group3_mRNA

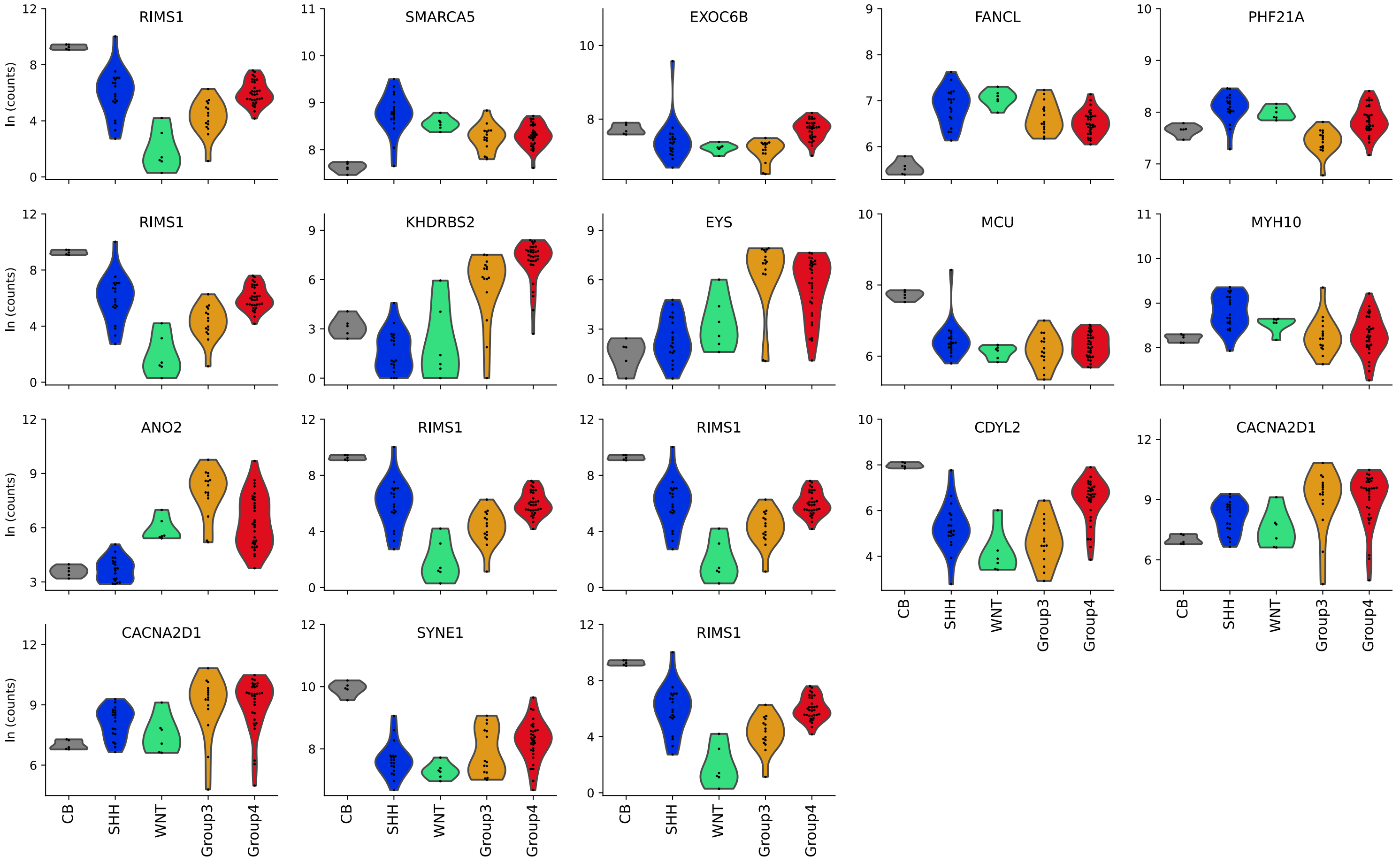

Figure S2D

Group4_circRNA

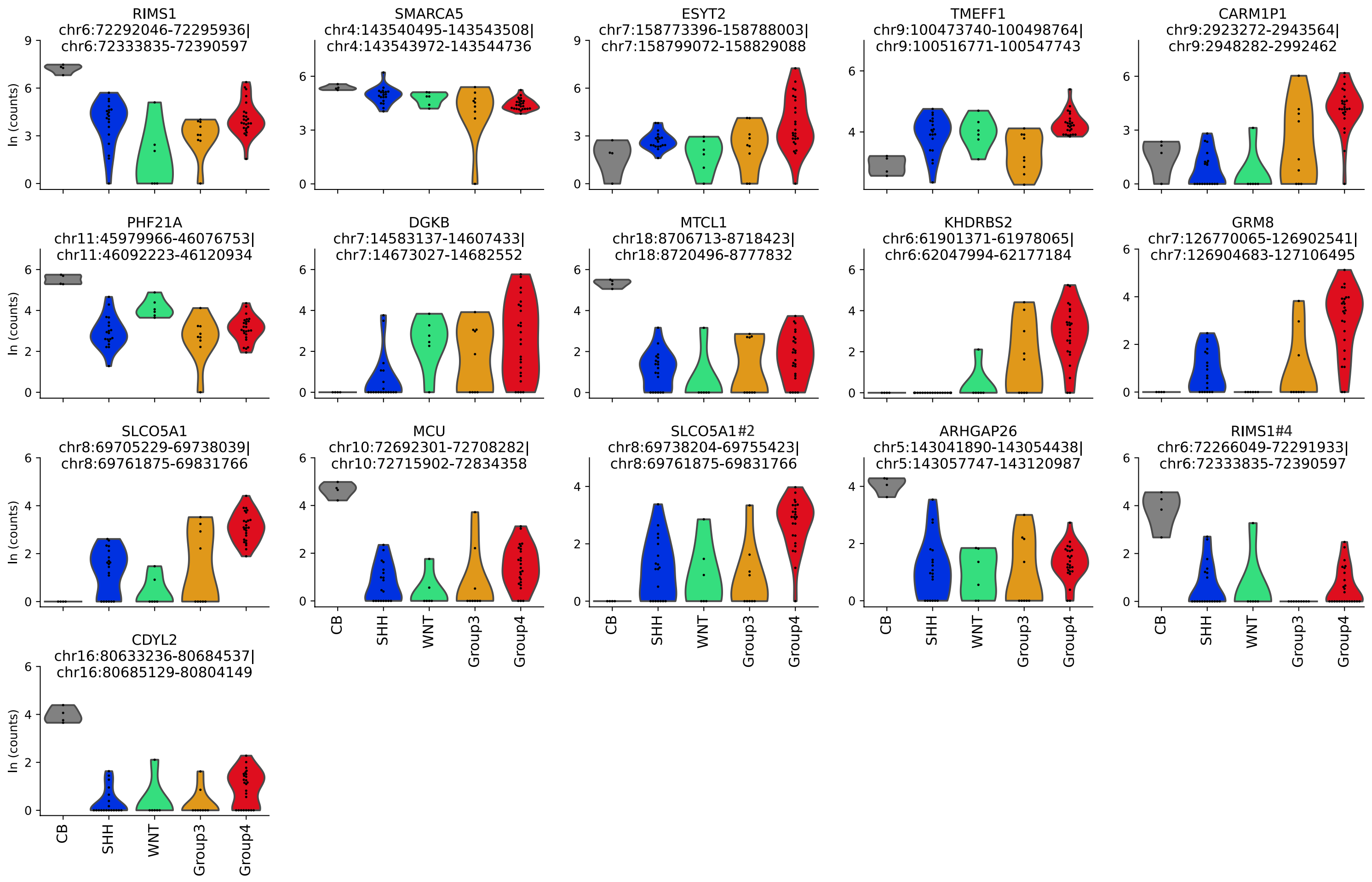

Group4_mRNA

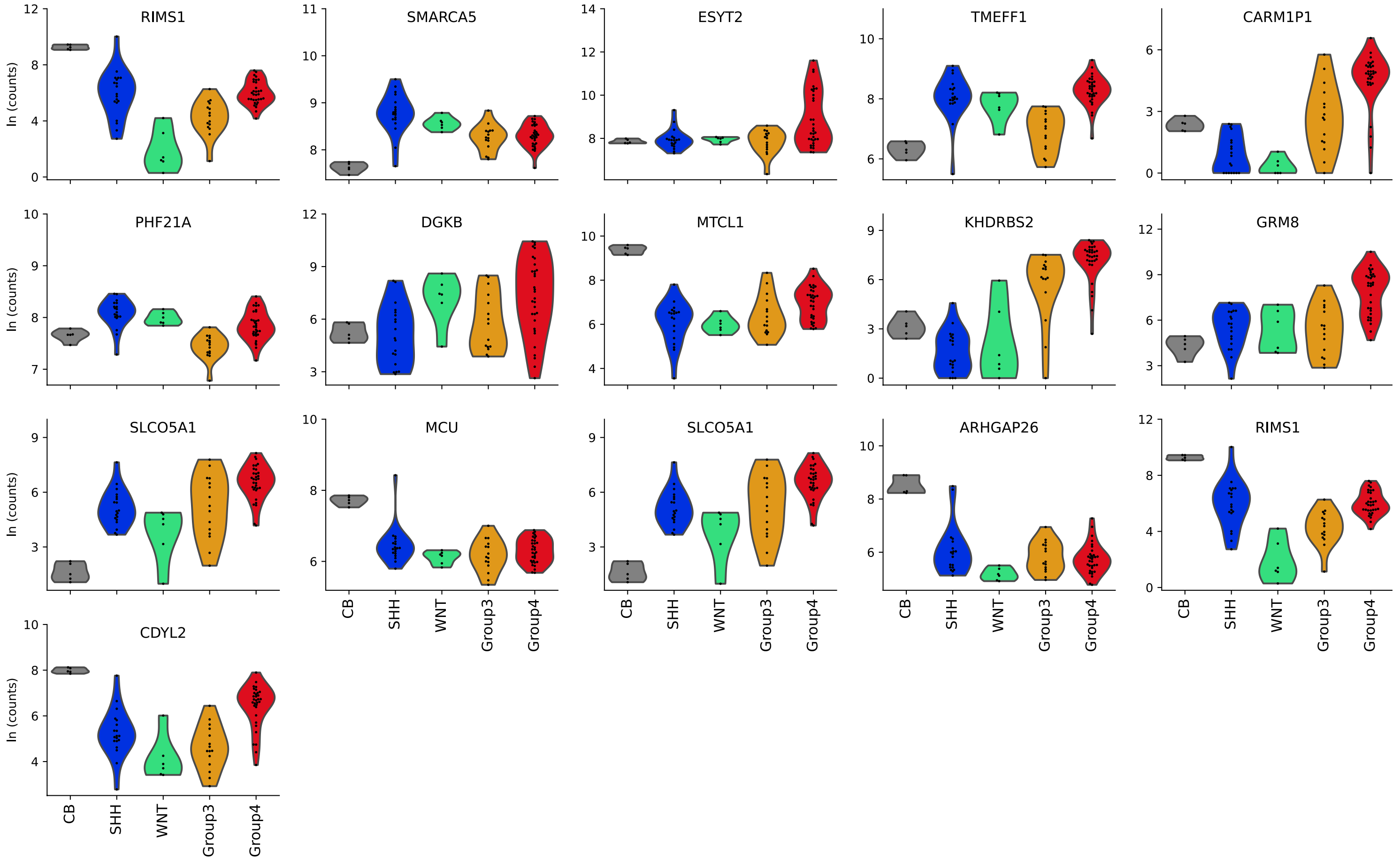

Figure S3

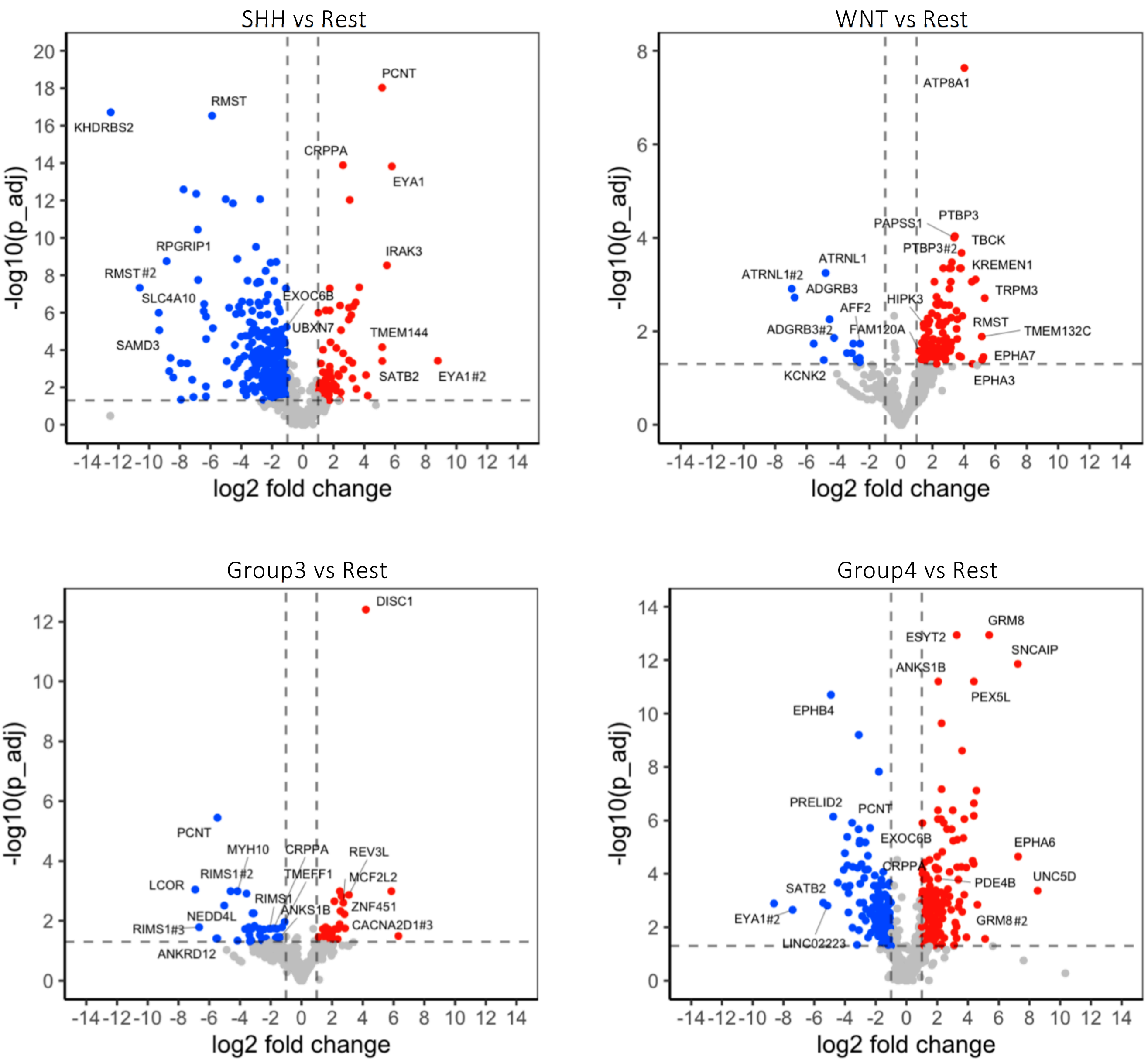
SHH vs Rest
-log10(p_adj)
log2 fold change
PCNT
RMST
KHDRBS2
CRPPA
EYA1
RPGRIP1
IRAK3
RMST#2
SLC4A10
EXOC6B
UBXN7
TMEM144
SAMD3
SATB2
EYA1#2
WNT vs Rest
ATP8A1
PTBP3
PAPSS1
TBCK
PTBP3#2
KREMEN1
ATRNL1
ATRNL1#2
ADGRB3
TRPM3
AFF2
HIPK3
RMST
ADGRB3#2
FAM120A
TMEM132C
EPHA7
KCNK2
EPHA3
Group3 vs Rest
DISC1
PCNT
MYH10
CRPPA
REV3L
RIMS1#2
TMEFF1
MCF2L2
LCOR
RIMS1
ANKS1B
ZNF451
NEDD4L
CACNA2D1#3
RIMS1#3
ANKRD12
Group4 vs Rest
GRM8
ESYT2
SNCAIP
ANKS1B
PEX5L
EPHB4
PRELID2
PCNT
EXOC6B
EPHA6
CRPPA
UNC5D
PDE4B
SATB2
EYA1#2
GRM8#2
LINC02223

Figure S4

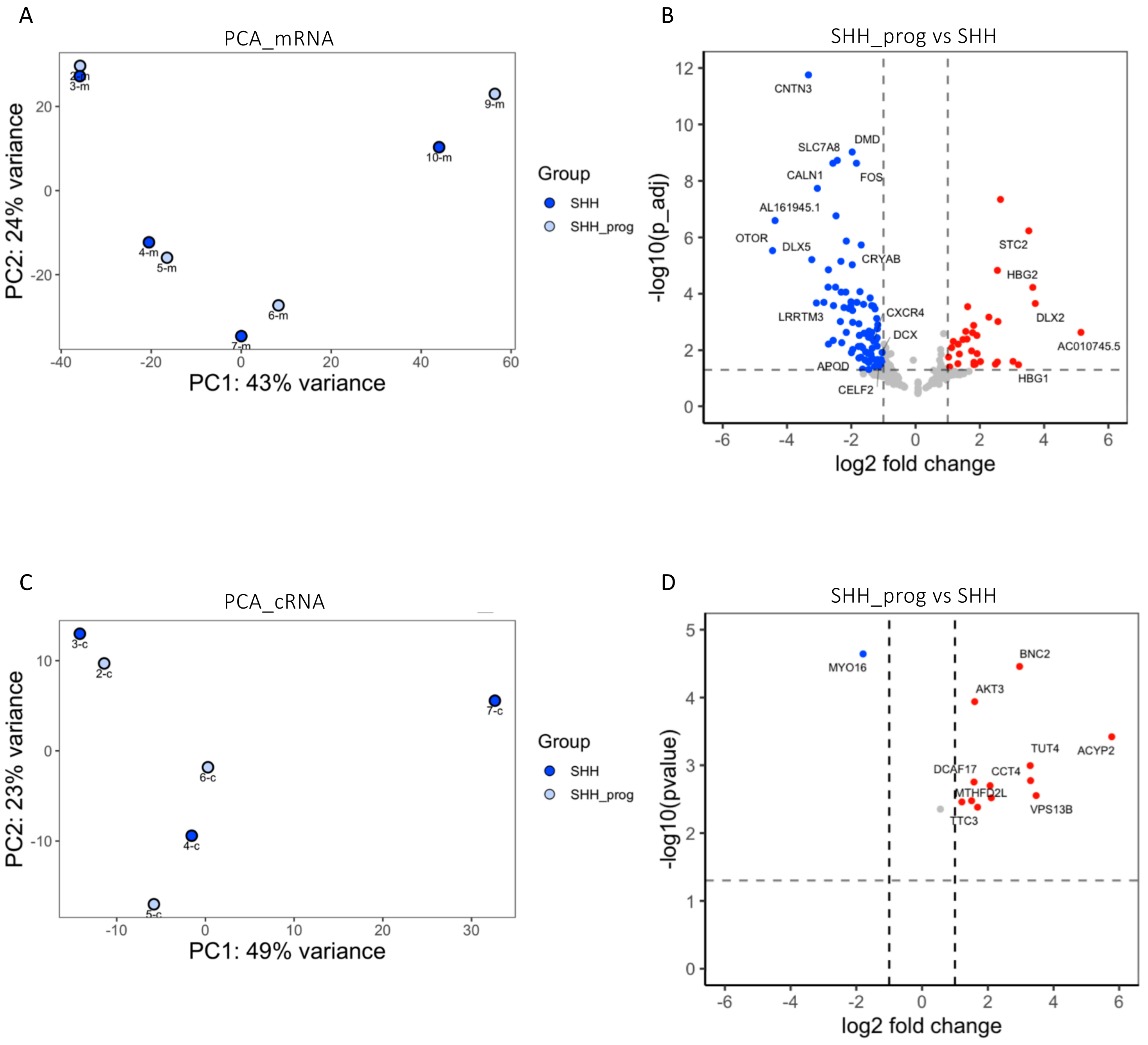
A
PCA_mRNA
PC2: 24% variance
PC1: 43% variance
2-m
3-m
9-m
10-m
4-m
5-m
6-m
7-m
Group
SHH
SHH_prog
B
SHH_prog vs SHH
-log10(p_adj)
log2 fold change
CNTN3
SLC7A8
DMD
CALN1
FOS
AL161945.1
OTOR
DLX5
CRYAB
LRRTM3
CXCR4
DCX
APOD
CELF2
STC2
HBG2
DLX2
AC010745.5
HBG1
C
PCA_cRNA
PC2: 23% variance
PC1: 49% variance
3-c
2-c
7-c
6-c
4-c
5-c
Group
SHH
SHH_prog
D
SHH_prog vs SHH
-log10(pvalue)
log2 fold change
MYO16
BNC2
AKT3
TUT4
ACYP2
DCAF17
CCT4
MTHFD2L
TTC3
VPS13B

Figure S5

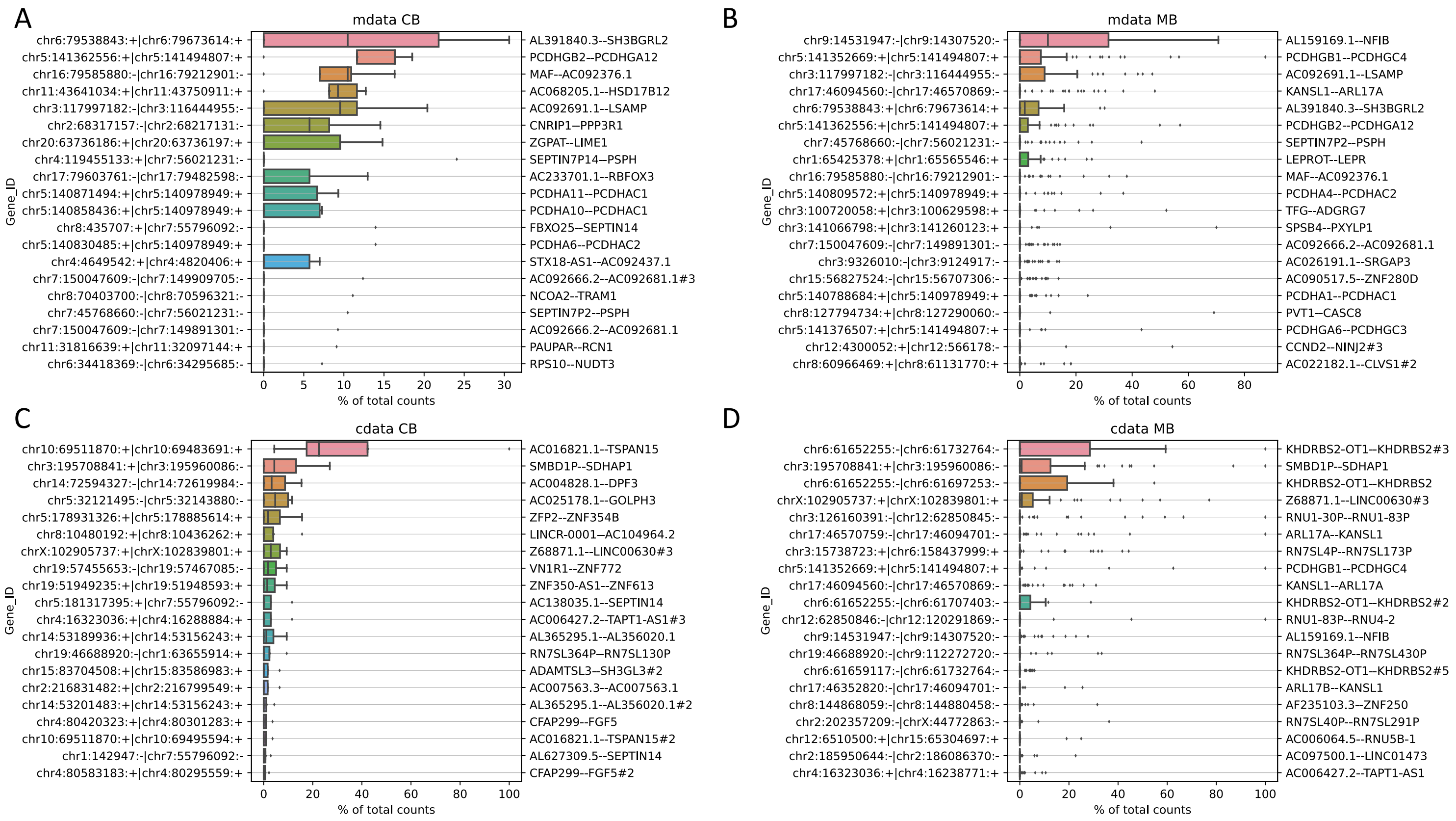
A
mdata CB
Gene_ID
% of total counts
chr6:79538843:+|chr6:79673614:+
chr5:141362556:+|chr5:141494807:+
chr16:79585880:-|chr16:79212901:-
chr11:43641034:+|chr11:43750911:+
chr3:117997182:-|chr3:116444955:-
chr2:68317157:-|chr2:68217131:-
chr20:63736186:+|chr20:63736197:+
chr4:119455133:+|chr7:56021231:-
chr17:79603761:-|chr17:79482598:-
chr5:140871494:+|chr5:140978949:+
chr5:140858436:+|chr5:140978949:+
chr8:435707:+|chr7:55796092:-
chr5:140830485:+|chr5:140978949:+
chr4:4649542:+|chr4:4820406:+
chr7:150047609:-|chr7:149909705:-
chr8:70403700:-|chr8:70596321:-
chr7:45768660:-|chr7:56021231:-
chr7:150047609:-|chr7:149891301:-
chr11:31816639:+|chr11:32097144:+
chr6:34418369:-|chr6:34295685:-
AL391840.3--SH3BGRL2
PCDHGB2--PCDHGA12
MAF--AC092376.1
AC068205.1--HSD17B12
AC092691.1--LSAMP
CNRIP1--PPP3R1
ZGPAT--LIME1
SEPTIN7P14--PSPH
AC233701.1--RBFOX3
PCDHA11--PCDHAC1
PCDHA10--PCDHAC1
FBXO25--SEPTIN14
PCDHA6--PCDHAC2
STX18-AS1--AC092437.1
AC092666.2--AC092681.1#3
NCOA2--TRAM1
SEPTIN7P2--PSPH
AC092666.2--AC092681.1
PAUPAR--RCN1
RPS10--NUDT3
0
5
10
15
20
25
30
B
mdata MB
Gene_ID
% of total counts
chr9:14531947:-|chr9:14307520:-
chr5:141352669:+|chr5:141494807:+
chr3:117997182:-|chr3:116444955:-
chr17:46094560:-|chr17:46570869:-
chr6:79538843:+|chr6:79673614:+
chr5:141362556:+|chr5:141494807:+
chr7:45768660:-|chr7:56021231:-
chr1:65425378:+|chr1:65565546:+
chr16:79585880:-|chr16:79212901:-
chr5:140809572:+|chr5:140978949:+
chr3:100720058:+|chr3:100629598:+
chr3:141066798:+|chr3:141260123:+
chr7:150047609:-|chr7:149891301:-
chr3:9326010:-|chr3:9124917:-
chr15:56827524:-|chr15:56707306:-
chr5:140788684:+|chr5:140978949:+
chr8:127794734:+|chr8:127290060:-
chr5:141376507:+|chr5:141494807:+
chr12:4300052:+|chr12:566178:-
chr8:60966469:+|chr8:61131770:+
AL159169.1--NFIB
PCDHGB1--PCDHGC4
AC092691.1--LSAMP
KANSL1--ARL17A
AL391840.3--SH3BGRL2
PCDHGB2--PCDHGA12
SEPTIN7P2--PSPH
LEPROT--LEPR
MAF--AC092376.1
PCDHA4--PCDHAC2
TFG--ADGRG7
SPSB4--PXYLP1
AC092666.2--AC092681.1
AC026191.1--SRGAP3
AC090517.5--ZNF280D
PCDHA1--PCDHAC1
PVT1--CASC8
PCDHGA6--PCDHGC3
CCND2--NINJ2#3
AC022182.1--CLVS1#2
0
20
40
60
80
C
cdata CB
Gene_ID
% of total counts
chr10:69511870:+|chr10:69483691:+
chr3:195708841:+|chr3:195960086:-
chr14:72594327:-|chr14:72619984:-
chr5:32121495:-|chr5:32143880:-
chr5:178931326:+|chr5:178885614:+
chr8:10480192:+|chr8:10436262:+
chrX:102905737:+|chrX:102839801:+
chr19:57455653:-|chr19:57467085:-
chr19:51949235:+|chr19:51948593:+
chr5:181317395:+|chr7:55796092:-
chr4:16323036:+|chr4:16288884:+
chr14:53189936:+|chr14:53156243:+
chr19:46688920:-|chr1:63655914:+
chr15:83704508:+|chr15:83586983:+
chr2:216831482:+|chr2:216799549:+
chr14:53201483:+|chr14:53156243:+
chr4:80420323:+|chr4:80301283:+
chr10:69511870:+|chr10:69495594:+
chr1:142947:-|chr7:55796092:-
chr4:80583183:+|chr4:80295559:+
AC016821.1--TSPAN15
SMBD1P--SDHAP1
AC004828.1--DPF3
AC025178.1--GOLPH3
ZFP2--ZNF354B
LINCR-0001--AC104964.2
Z68871.1--LINC00630#3
VN1R1--ZNF772
ZNF350-AS1--ZNF613
AC138035.1--SEPTIN14
AC006427.2--TAPT1-AS1#3
AL365295.1--AL356020.1
RN7SL364P--RN7SL130P
ADAMTSL3--SH3GL3#2
AC007563.3--AC007563.1
AL365295.1--AL356020.1#2
CFAP299--FGF5
AC016821.1--TSPAN15#2
AL627309.5--SEPTIN14
CFAP299--FGF5#2
0
20
40
60
80
100
D
cdata MB
Gene_ID
% of total counts
chr6:61652255:-|chr6:61732764:-
chr3:195708841:+|chr3:195960086:-
chr6:61652255:-|chr6:61697253:-
chrX:102905737:+|chrX:102839801:+
chr3:126160391:-|chr12:62850845:-
chr17:46570759:-|chr17:46094701:-
chr3:15738723:+|chr6:158437999:+
chr5:141352669:+|chr5:141494807:+
chr17:46094560:-|chr17:46570869:-
chr6:61652255:-|chr6:61707403:-
chr12:62850846:-|chr12:120291869:-
chr9:14531947:-|chr9:14307520:-
chr19:46688920:-|chr9:112272720:-
chr6:61659117:-|chr6:61732764:-
chr17:46352820:-|chr17:46094701:-
chr8:144868059:-|chr8:144880458:-
chr2:202357209:-|chrX:44772863:-
chr12:6510500:+|chr15:65304697:+
chr2:185950644:-|chr2:186086370:-
chr4:16323036:+|chr4:16238771:+
KHDRBS2-OT1--KHDRBS2#3
SMBD1P--SDHAP1
KHDRBS2-OT1--KHDRBS2
Z68871.1--LINC00630#3
RNU1-30P--RNU1-83P
ARL17A--KANSL1
RN7SL4P--RN7SL173P
PCDHGB1--PCDHGC4
KANSL1--ARL17A
KHDRBS2-OT1--KHDRBS2#2
RNU1-83P--RNU4-2
AL159169.1--NFIB
RN7SL364P--RN7SL430P
KHDRBS2-OT1--KHDRBS2#5
ARL17B--KANSL1
AF235103.3--ZNF250
RN7SL40P--RN7SL291P
AC006064.5--RNU5B-1
AC097500.1--LINC01473
AC006427.2--TAPT1-AS1
0
20
40
60
80
100

Figure S6

Original gel electrophoresis for **Figure 3**

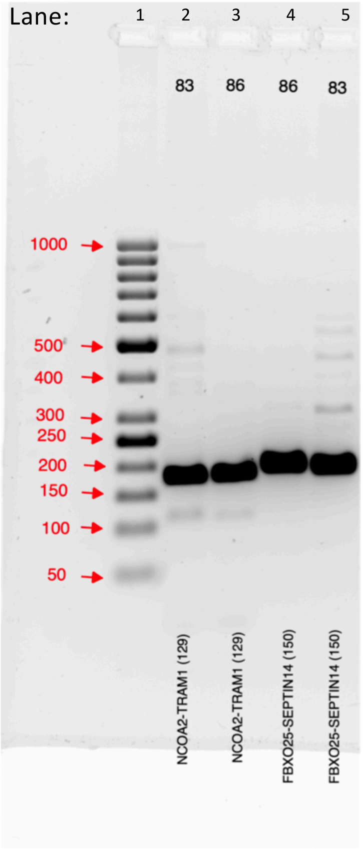

Supplement: Supplementary file 1 [file cancers-14-03134-s001.zip › Supplementary Figures S1--S6.pdf]
